# Acute glucose stimulation drives coordinated translational reprogramming in primary pancreatic islets: from global remodeling to fine-tuned insulin synthesis

**DOI:** 10.3389/fendo.2026.1834362

**Published:** 2026-06-18

**Authors:** Yiqing Wang, Chunyang Shi, Yao Liu, Wenli Feng, Ming Liu, Xiaoxi Xu

**Affiliations:** Department of Endocrinology and Metabolism, Tianjin Medical University General Hospital, Tianjin, China

**Keywords:** insulin biosynthesis, pancreatic β cells, ribosome profiling, translational regulation, type 2 diabetes

## Abstract

**Background:**

Pancreatic beta cells must rapidly escalate protein synthesis to maintain systemic glucose homeostasis. While the transcriptional responses are well characterized, the immediate translational dynamics governing this adaptive phase remain poorly defined.

**Methods:**

We performed high-resolution ribosome profiling (Ribo-seq) on primary mouse islets under acute low-glucose (2.5 mM) and high-glucose (25 mM) conditions and integrated analysis of the differential translation, functional enrichment, translational efficiency (TE), and ribosome kinetics. The protein levels and mRNA expression were validated using Western blot and quantitative PCR (qPCR), respectively.

**Results:**

We identified extensive translational reprogramming involving 1, 680 differentially translated genes. High glucose triggered a significant upregulation of immediate early genes (e.g., *Fos* and *Nr4a1*) and a concurrent inhibition of stress-related genes (e.g., *Ddit3* and *Trib3*). On the other hand, beta cells prioritized the synthesis of cytosolic ribosomal proteins and elongation factors to expand the biosynthetic machinery. This was coordinated with a scale-up of the downstream secretory pathway (e.g., *Sec61a1*) and a metabolic realignment, characterized by the translational upregulation of mitochondrial enzymes (e.g., *Cs* and *Fh1*) despite the relative suppression of mitochondrial biogenesis genes. Furthermore, TE analysis revealed that several genes were regulated independent of their mRNA levels, such as *Rpl3* and *Atf4*. Finally, kinetic analysis suggested that high glucose affected the ribosome occupancy density and distribution on specific transcripts, such as *Ins1*.

**Conclusion:**

Our research characterizes the translatome as a dynamic regulator of the glucose response. By revealing these rapid translational nodes, we provide potential targets to restore the insulin synthetic capacity and secretory function in T2DM, offering a mechanistic framework for the development of therapies centered on preserving β-cell proteostasis.

## 1 Introduction

Type 2 diabetes mellitus (T2DM) is a globally escalating metabolic crisis, characterized by chronic hyperglycemia and a rising incidence of complications, including cardiovascular disease, nephropathy, and neuropathy, which contribute significantly to global mortality and economic burden (1). At its core, the pathophysiology of T2DM involves a combination of insulin resistance and inadequate insulin secretion from pancreatic beta cells. However, the disease is highly heterogeneous, and the clinical efficacy of the existing interventions varies considerably among patients (2). This complexity necessitates the continuous development of precision medicine-based therapeutic strategies and the acceleration of their transition from bench to bedside (1).

The ability of beta cells to sense glucose stimulation and mount a prompt, appropriate insulin secretion is fundamental to systemic glycemic control (3). While the first phase of glucose-stimulated insulin secretion (GSIS) relies on the exocytosis of stored insulin granules, the *de novo* synthesis of preproinsulin plays a more critical role in the second phase. An early transcriptomic study in glucose-stimulated Min6 cells identified differentially expressed genes (DEGs) involved in the secretory pathway, metabolism, cell signaling, and transcription (4). Notably, multiple components of the endoplasmic reticulum (ER) translocon were significantly upregulated. Given that the co-translational translocation of preproinsulin into the ER constitutes the first rate-limiting step in insulin production at the protein level, these findings highlight the importance of translational control in β-cell function. Indeed, our previous research and that of others have demonstrated that the loss of these components impairs insulin biosynthesis and disrupts systemic glucose homeostasis in mice (5–9).

Because proteins are the direct functional effectors of cellular processes, the transcriptome only partially reflects the extent of reprogramming. Recent works have begun to unravel the translational dynamics of beta cells. One research performed polysome profiling in a human β-cell line (EndoC-H1), which revealed that the mammalian target of rapamycin (mTOR) and eIF2α pathways independently upregulate the translation machinery during acute glucose stimulation (10). This mechanism facilitates the adaptation of beta cells to impending metabolic demands. Similarly, another investigation in Min6 cells found that numerous messenger RNAs (mRNAs) associated with oxidative stress exhibited altered translation rates under high glucose (11). Collectively, these studies indicate that high glucose triggers a highly dynamic and hierarchical reprogramming of the translational landscape. However, identifying the specific glucose-responsive translatome and understanding how these genes coordinate to maintain β-cell function remain a largely open question, particularly in primary islets, which more closely reflect physiological conditions.

While polysome profiling has traditionally been a classic method for assessing translation, it lacks positional information and requires substantial quantities of cells. In our study, we employed ribosome profiling (Ribo-seq) analysis in primary mouse islets. This technique captures ribosome-protected mRNA fragments (RPFs), providing a nucleotide resolution- and site-specific snapshot of dynamically translating ribosomes. This approach allows for a more precise quantification of the translational efficiency (TE) at a genome-wide scale and can uncover regulatory features—such as translational pausing and elongation kinetics—that are invisible to conventional methods (12).

By comparing the Ribo-seq landscapes under low- and high-glucose conditions, we aimed to define the glucose-responsive genes at the translational level. Our findings advance the understanding of β-cell physiology and T2DM pathogenesis, as well as the development of potential innovative therapeutic strategies.

## 2 Materials and methods

### 2.1 Mouse islet isolation

Pancreatic islets were isolated from 6- to 8-week-old male mice on a C57BL/6J background by collagenase digestion and recovered in complete RPMI 1640 medium containing 11.1 mM glucose overnight. The following day, the islets from each individual mouse were equally distributed into two glucose conditions by handpicking and were pre-incubated in glucose-free RPMI 1640 medium containing 10% fetal bovine serum for 4 h. A glucose stock solution was spiked in reaching the final concentration of either 2.5 or 25 mM and then the culture continued for another 2 h. Cycloheximide at 100 μg/ml was added to each sample at the last 15 min before collection to fix the ribosomes. The islets were then pelleted and snap-frozen in liquid nitrogen. Three biological replicates were prepared for each group. A total of 24 animals were used in three independent experiments, and islets from seven to nine mice were pooled as a biological replicate to obtain sufficient material. Overall, approximately 500–600 islets were used for each Ribo-seq library. All sequencing and downstream analyses were performed independently. All animal procedures were approved by the Laboratory Animal Management and Use Committee (IACUC) of Tianjin Medical University (approval no. TMUaMEC2025030).

### 2.2 Ribosome profiling and RNA-seq

Cell lysates were treated with RNase I to digest the RNA without ribosome protection. Monosomes were isolated by size exclusion chromatography with MicroSpin S-400 HR columns and PAGE purification. Library construction was prepared using the Multiplex Small RNA Library Prep Set for Illumina (Set1; NEB, Ipswich, MA, USA) with ribosomal RNA (rRNA) decontamination using an rRNA depletion kit (Qiagen, Hilden, Germany). Samples were subjected to Illumina sequencing using SE50 (single-end 50 nt). Clean data with high quality were generated for downstream analyses, including the removal of adapters and low-quality sequences (reads with 5′ adapter, reads without 3′ adapter or insert sequence, reads with more than 10% N, and reads with more than 30% nucleotides with *Q*_phred_ ≤ 20) (12). rRNA and transfer RNA (tRNA) reads were also filtered by aligning to SILVA and the Rfam database using Bowtie (version 1.1.2) with no mismatch allowed. Clean reads were mapped to the mouse genome using TopHat2 (2.0.12). Quantification of gene expression was performed with HTSeq (0.9.1), and RPKM (reads per kilobase of transcript per million mapped reads) values were generated for each gene.

For bulk RNA sequencing (RNA-seq), islets from the same culture conditions were subjected to RNA extraction using the Qiagen RNeasy kit. Libraries were constructed using the Fast RNA-seq Lib Prep Kit v2 (cat. RK20306; ABclonal) and sequenced on an Illumina platform using sequencing-by-synthesis. Raw reads were processed with fastp for quality control. Clean reads were aligned to the reference genome using HISAT2, and gene-level read counts were obtained with featureCounts.

### 2.3 Western blot

A parallel set of islets incubated in 2.5 or 25 mM glucose was lysed in RIPA buffer on ice for 15 min and centrifuged at 12, 000 rpm at 4°C for 15 min. The supranatants were boiled in a sample buffer containing 100 mM dithiothreitol (DTT) for 5 min. Equal amounts of protein samples were loaded onto 4%–12% NuPage gradient gels (Thermo Fisher Scientific, Waltham, MA, USA), transferred into a nitrocellulose membrane (BioTrace NT Nitrocellulose Membrane, catalog 66485; PALL, Port Washington, NY, USA), and blotted with indicated primary antibodies followed by secondary antibodies conjugated with horseradish peroxidase (HRP). The antibodies used in this study were as follows: anti-proinsulin (CCI-17) (cat. no. NB100-73013; Novus Biologicals, Centennial, CO, USA), anti-insulin B-chain (homemade), anti-β-actin (cat. no. UM4001; UTIBODY, Tianjin Youkang Biotechnology Co., Ltd., Tianjin 300192, China), anti-p-eIF2α (cat. no. 9721S; Cell Signaling Technology, Danvers, MA, USA), cFos (cat. no. 2250; Cell Signaling Technology), CHOP (cat. no. A0221; ABclonal, Woburn, MA, USA), and pS6 (cat. no. 4858; Cell Signaling Technology).

### 2.4 Bioinformatics analysis and data visualization

Differential expression analysis between the low- and high-glucose conditions was carried out using the DESeq2 R package (v1.14.1). An absolute fold change of 1.5 and a *p*-value of 0.05 were set as the thresholds for significant differential expression (|log_2_FC| > 0.58, with *p* < 0.05). These genes were subjected to functional enrichment analysis, including Gene Ontology (GO) and Kyoto Encyclopedia of Genes and Genomes (KEGG) pathways, using the clusterProfiler R package. Gene set enrichment analysis (GSEA) was also conducted against the MSigDB database to minimize threshold bias and capture subtle systemic changes.

The heatmaps for functional modules were generated using the ComplexHeatmap R package. The gene lists originated from the GSEA datasets and were supplemented manually according to curated nomenclature patterns. Per-gene log_2_FC values were visualized as right-side bar plot annotations. An enrichment map (EMAP) was generated with the emapplot function to explore the relationships between the enriched pathways, and category–gene network plots (cnetplot) were used to illustrate the relationships between specific genes and their associated biological functions.

To visualize the distribution of the RPFs along individual mRNAs with the GenomicAlignments R package, normalized reads were averaged from uniquely mapped BAM files across three biological replicates. The genomic coordinates of the coding DNA sequence (CDS) for the longest transcript of specific genes were retrieved. Nucleotide signals were reversed for genes located on the minus strand. The final ribosome occupancy profiles were visualized as overlapping area plots using the ggplot2 R package.

To elucidate the underlying mechanisms of translational regulation, TE was calculated as the ratio of the RPKM in Ribo-seq to the RPKM in RNA-seq. A nine-quadrant plot was generated by correlating the log_2_FC values accordingly. Functional enrichment for each sub-cluster was analyzed as mentioned above.

### 2.5 Statistical analysis

Significance for physiological experiments was assessed with the Student’s *t*-test and one-way ANOVA using GraphPad Prism 10 software. Data are presented as the mean ± SEM. *P*-values <0.05 were considered as statistically significant.

## 3 Results

### 3.1 Ribo-seq reveals global dynamic mRNA translational regulation under acute glucose stimulation

**Figure 1:**
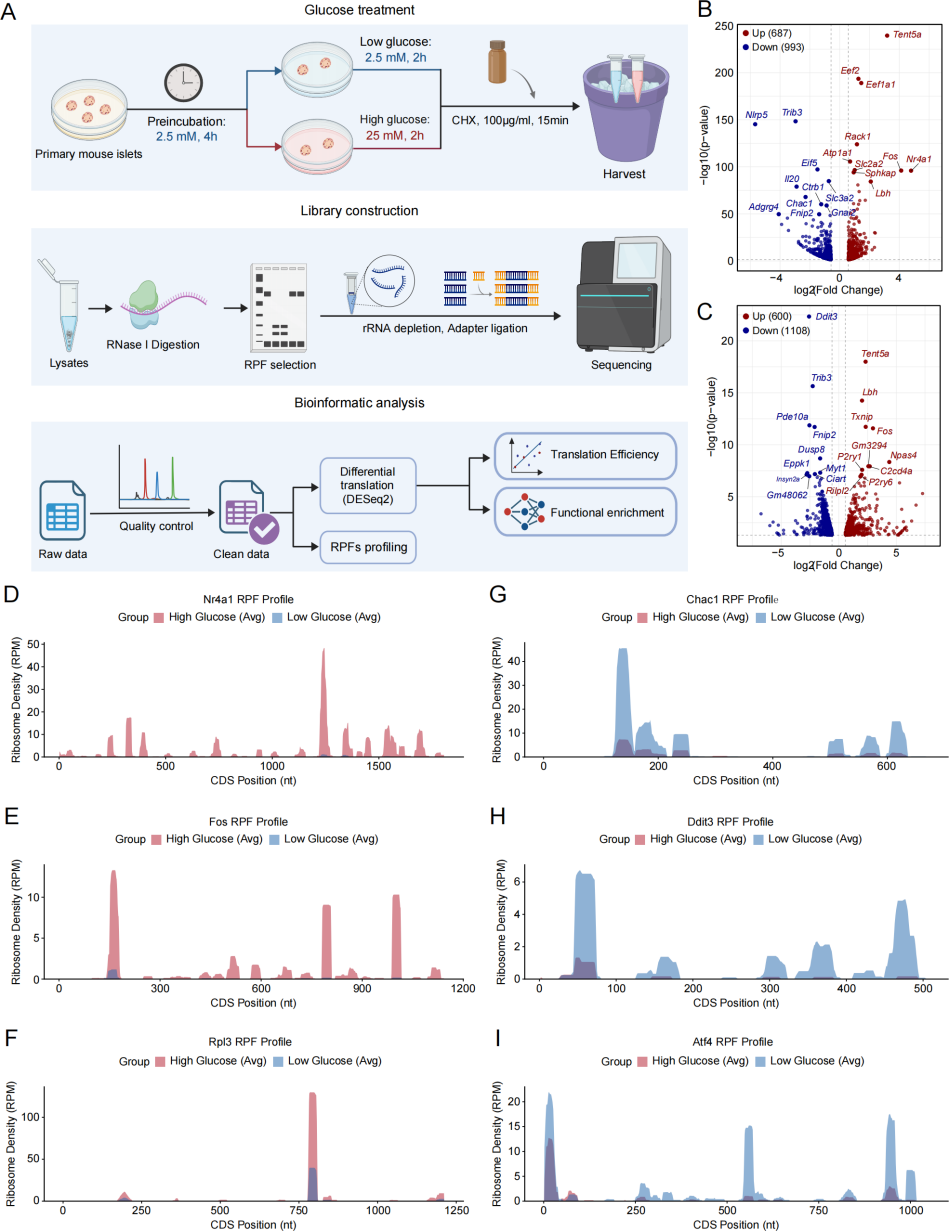
Ribosome profiling (Ribo-seq) pipeline, volcano plots, and representative ribosome-protected mRNA fragment (RPF) plots. **(A)** Illustrative diagram of the Ribo-seq process used to map the experimental design of acute high-glucose stimulation in mouse primary pancreatic islets and the bioinformatics analysis process of the sequencing results. **(B, C)** Volcano plots showing the differentially expressed genes (DEGs) at the translational level **(B)** and the transcriptional level **(C)**. Red indicates upregulation, blue indicates downregulation, and numbers represent the counts of DEGs (fold change threshold >1.5 and *p*-value <0.05). (**D**–**I**) Differential distribution curves of ribosomal relative density (in reads per million mapped reads, RPM) across the CDS position (nucleotides) of translational DEGs, which are well-known targets of glucose.

To identify glucose-regulated genes, primary mouse islets were incubated in RPMI 1640 medium containing either 2.5 or 25 mM glucose for 2 h and subjected to sequencing (**Figure 1A**). Differential expression analysis using DESeq2 identified 1, 680 genes exhibiting significantly altered translation levels under high-glucose conditions compared with low glucose (687 upregulated and 993 downregulated, defined by a fold change threshold >1.5 and *p* < 0.05) (**Figure 1B**). Data also revealed 1, 708 DEGs in bulk RNA-seq, with 600 upregulated and 1, 108 downregulated using the same criteria (**Figure 1C**).

Many translational DEGs are well-known targets of glucose. Most strikingly, several immediate early genes (IEGs) ranked at the top of this list, which typically function as master regulators of downstream transcriptional changes, including *Nr4a1* (log_2_FC = 4.79), *Nr4a2* (log_2_FC = 1.67), *Fos* (log_2_FC = 4.13), *Fosl2* (log_2_FC = 1.81), *Crem* (log_2_FC = 1.48), *Npas4* (log_2_FC = 2.33), *Irs2* (log_2_FC = 1.39), and *Sik1* (log_2_FC = 1.64). Conversely, the genes related to stress, apoptosis, and cell cycle arrest were downregulated, including *Trib3* (log_2_FC = −2.96), *Ddit3* (log_2_FC = −2.25), *Atf4* (log_2_FC = −1.24), and *Chac1* (log_2_FC = −2.29) (**Supplementary Table 1**). To determine whether these translational shifts stem from an altered ribosome occupancy, we directly visualized the RPFs across these genes. Consistent with our differential analysis, a marked divergence in RPF density was observed between the low- and high-glucose conditions (**Figures 1D–I**).

### 3.2 High glucose triggers a dose-dependent translational scaling of insulin biosynthesis

**Figure 2:**
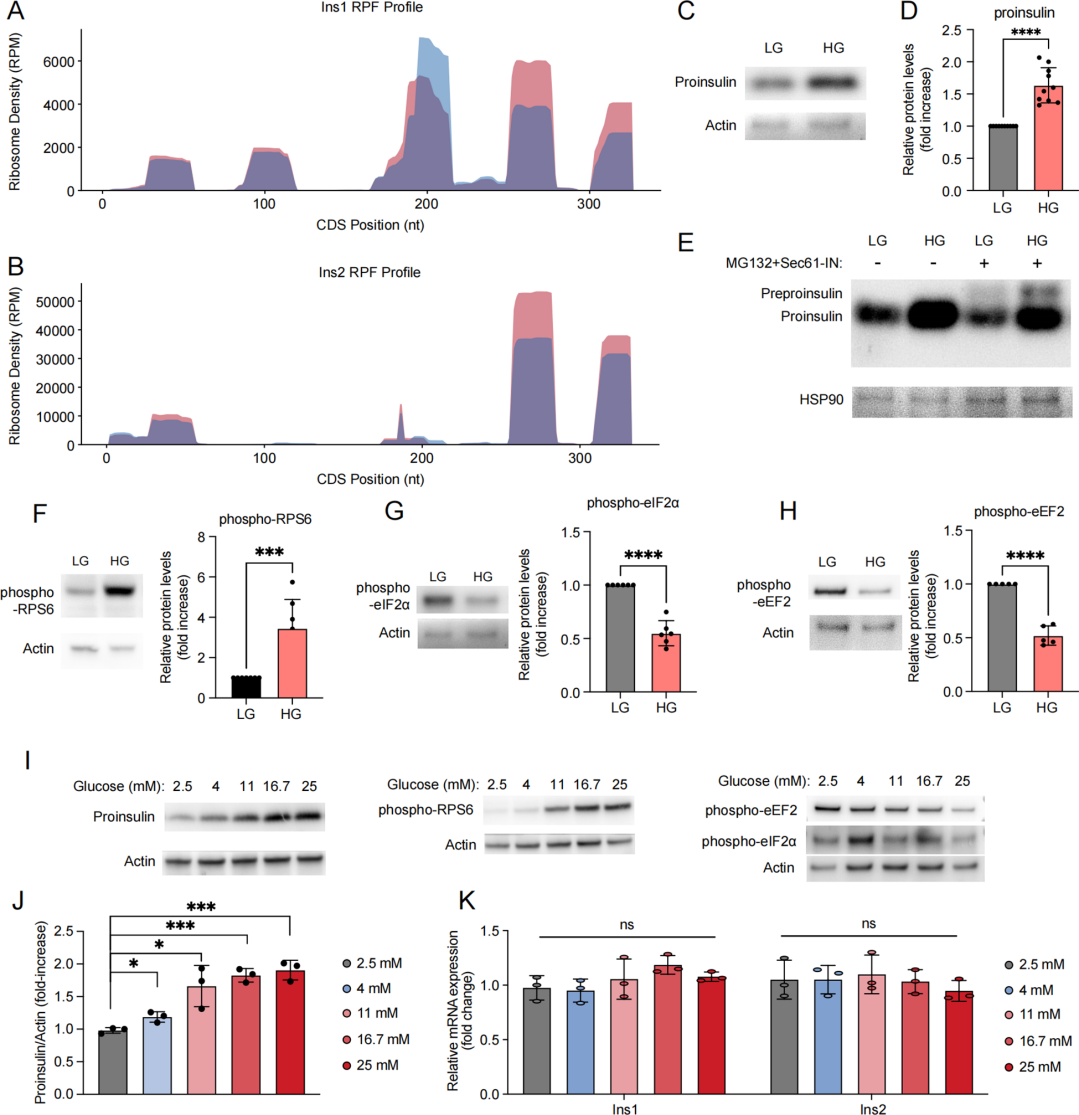
Western blot validation of proinsulin, preproinsulin, p-RPS6, p-eIF2α, and p-eEF2. **(A, B)** Differential distribution curves of ribosomal relative density (in reads per million mapped reads, RPM) across the CDS position (nucleotides) of Ins1 **(A)** and Ins2 **(B)**. **(C, D)** Western blots and quantification of proinsulin from low- and high-glucose stimulation in primary mouse islets for 2 h using actin as the loading control. Values are the mean ± SD (*n* = 10 biological replicates). **(E)** Western blots of preproinsulin and proinsulin from low- and high-glucose stimulation for 2 h, without or with the endoplasmic reticulum (ER) translocation inhibitor and proteasome inhibitor MG132 for the last 30 min using actin as the loading control. **(F–H)** Western blots and quantification of phospho-RPS6 **(F)**, phospho-eIF2α **(G)**, and phospho-eEF2 **(H)** in primary mouse islets treated with low and high glucose for 2 h. Values are the mean ± SD (n = 4-6 biological replicates). **(I)** Western blots of proinsulin, phospho-RPS6, phospho-eIF2α, and phospho-eEF2 from a broader glucose range (2.5, 4, 11, 16.7, and 25 mM) stimulation in primary mouse islets for 2 h. **(J)** Quantification of proinsulin from **(I)**. **(K)** Quantitative analysis of Ins1 and Ins2 expression via real-time quantitative PCR (RT-qPCR) in primary mouse pancreatic islets stimulated with a broad range of glucose range (2.5, 4, 11, 16.7, and 25 mM) stimulation for 2 h. *: P < 0.05; ***: P < 0.001; ****: P < 0.0001; ns: not significant.

Glucose responsiveness and insulin biosynthesis are the defining features of pancreatic beta cells. Our Ribo-seq data showed an increased ribosome occupancy on Ins1 and Ins2 mRNAs (log_2_FC = 0.15 and 0.34, respectively) (**Supplementary Table 2**). Interestingly, we detected a prominent RPF peak mapping to the coding sequence corresponding to amino acids (AA) 56–72 of preproinsulin-1. The magnitude of this peak was significantly reduced relative to the downstream ribosome occupancy under high-glucose conditions. Similar kinetic signatures of the altered RPF distribution were also observed for several other genes encoding signal peptide-containing proteins, although the effect was less pronounced for Ins2 (**Figures 2A, B**).

Western blotting confirmed elevated levels of proinsulin and preproinsulin under high-glucose conditions (**Figures 2C–E**). This was accompanied by the phosphorylation of RPS6 (a marker of mTORC1 activation) and the dephosphorylation of eIF2α and eEF2 (**Figures 2F–H**). These events reflect a coordinated relief of translational “brakes” on initiation and elongation, providing a mechanistic basis for the acute phase captured in our Ribo-seq.

To ensure the biological relevance of our findings, we extended the validation across a broader glucose range (i.e., 2.5, 4, 11, 16.7, and 25 mM). We observed a dose-dependent increase in the proinsulin output and the RPS6 phosphorylation (**Figure 2I**). Notably, while the corresponding insulin mRNA levels remained remarkably stable across these concentrations as measured by real-time quantitative PCR (RT-qPCR), underscoring the predominant role of translational control in the acute glucose response (**Figures 2J, K**).

### 3.3 Translation and RNA processing pathways are significantly enriched under high glucose within a 2-h time frame

**Figure 3:**
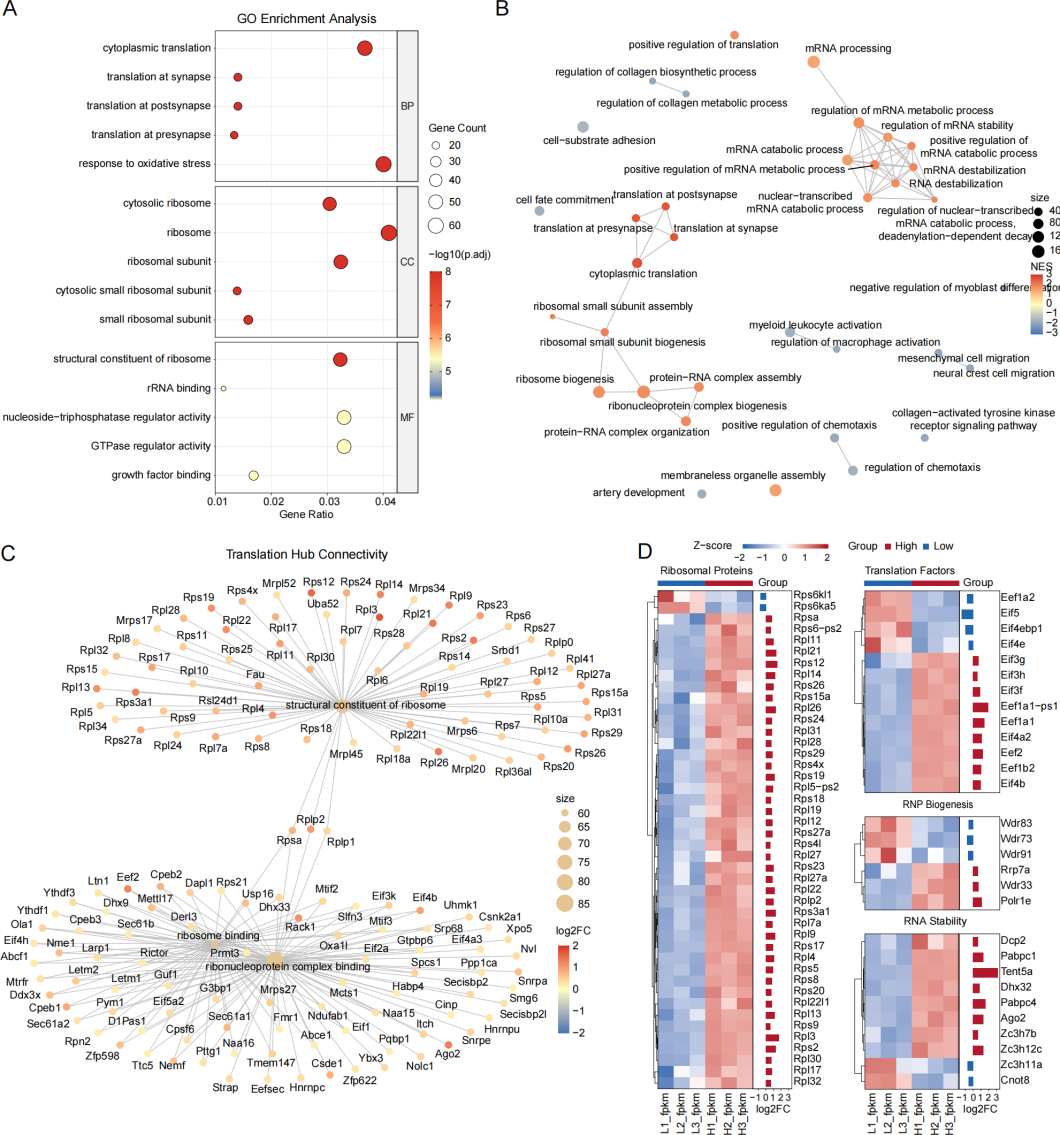
Translation regulation of the translation-related genes. **(A)** Bubble plots illustrating the enrichment of Gene Ontology (GO) terms for differentially expressed genes associated with cytoplasmic translation, ribosome biogenesis, and ribosome assembly under high-glucose stimulation. The color of each bubble represents the adjusted −log10 (*p*_adj_.), the size of the bubble corresponds to the gene count, and the location of the bubble denotes the GO category (i.e., biological process, cellular component, and molecular function) and gene count (the proportion of genes in the subset mapped to that pathway). **(B)** Enrichment map of the global pathway network. The color of each bubble represents the normalized enrichment score (NES; *p*_adj._), and the size of the bubble corresponds to the gene count. **(C)** Translation hub connectivity map of the genes that contribute to structural constituent and ribonucleoprotein complex binding pathways. The color of each bubble represents the log_2_FC, and the size of the bubble corresponds to the contributions score. **(D)** Heatmap of the genes in pathways associated with ribosomal proteins, translation factors, ribonucleoprotein (RNP) biogenesis, and RNA stability. Red indicates upregulation, while blue indicates downregulation. The color gradient of each gene corresponds to its scaled *Z* score. Bar length reflects the log_2_FC.

GO analysis revealed a profound impact on the translational machinery, pointing to a centralized mobilization of the networks related to cytoplasmic translation, ribosome biogenesis, and ribosome assembly (**Figure 3A**). The EMAP showed a dominant “translation hub” where nodes representing “cytoplasmic translation” [normalized enrichment score (NES) = 2.60] and “ribonucleoprotein complex biogenesis” (NES = 1.78) were clustered together (**Figure 3B**).

The translation hub connectivity map (**Figures 3C, D**) further resolves the contributions of specific genes to the structural constituent and ribonucleoprotein complex binding pathways. Specifically, *Rpl3*, a key structural component of the large ribosomal subunit, displayed the highest induction (log_2_FC = 1.72), while *Rps12* was the most upregulated within the small subunit (log_2_FC = 1.55). It also underscores the central role of the key upregulated genes such as *Rplp2* (log_2_FC = 1.02) and *Rpsa* (log_2_FC = 0.78) in reinforcing the ribosomal framework. Key translation factors such as *Eef1a1* (log_2_FC = 1.45), *Eef2* (log_2_FC = 1.26), and *Eif4b* (log_2_FC = 0.98) were scaled up at the same time, favoring the molecular environment for the recruitment and movement of the translation complex.

Furthermore, the “Rescue of Stalled Ribosome” category showed the upregulation of *Rack1*, *Nemf*, and *Zfp598. Rack1*, a ribosomal scaffolding protein that integrates multiple signaling pathways, was upregulated approximately twofold (log_2_FC = 1.16). *Nemf* and *Zfp598*, which are critical components of the ribosome quality control (RQC) system (with log_2_FC values of 0.62 and 0.66, respectively), also increased over 1.5-fold. These results indicate that islets exhibit an increased translational signature of the components involved in the clearance of stalled ribosomes and recycling subunits, potentially preventing proteotoxic stress.

Another highly interconnected hub was composed of “positive regulation of mRNA metabolic process” (NES = 1.88) and mRNA turnover pathways (deadenylation-dependent decay: NES = 1.92; mRNA destabilization: NES = 1.89). *Tent5a* (log_2_FC = 3.19), *Pabpc4* (log_2_FC = 1.58), and *Pabpc1* (log_2_FC = 1.30) were upregulated coordinately, suggesting a critical regulation mechanism through maintenance of the stability of the existing mRNA transcripts beyond simply expanding ribosome biogenesis to optimize the protein output.

### 3.4 Coordinated translational scaling of the secretory machinery under acute high-glucose stimulation

**Figure 4:**
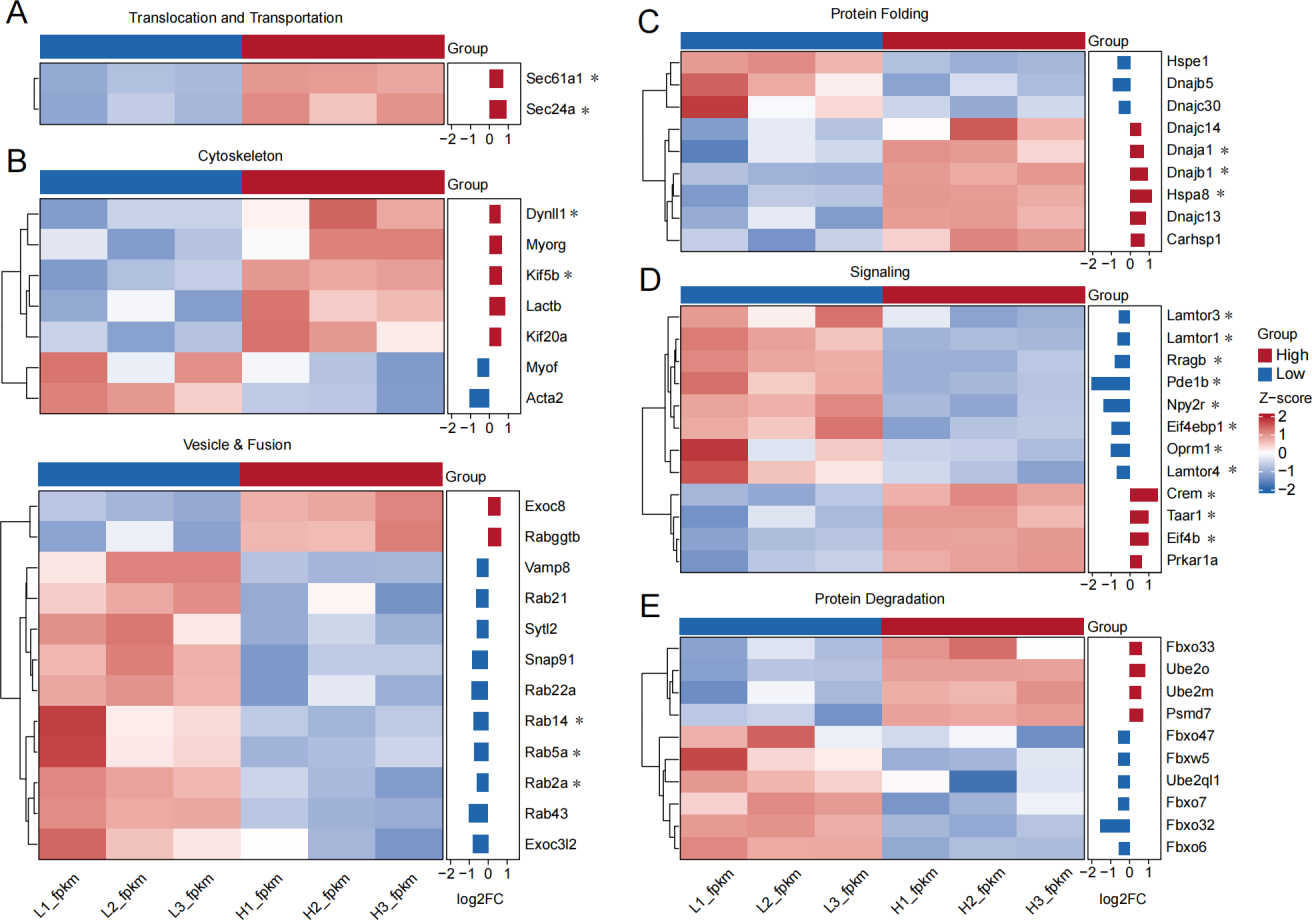
Translational regulation of the insulin secretory pathway. Heatmap of genes involved in pathways related to translocation and transportation **(A)**, cytoskeleton and vesicle & fusion **(B)**, protein folding **(C)**, signaling **(D)**, and protein degradation **(E)**. The color gradient of each gene indicates its scaled Z score. Bar length represents the log_2_FC. *: P < 0.05.

Systemic investigation showed that acute high glucose also orchestrates a coordinated but modest upregulation of the secretory logistical framework (**Figure 4A**; **Supplementary Table 2**). The simultaneous activation of translocation in response to the high demand for insulin synthesis was evidenced by a significant translational upregulation of *Sec61a1* (log_2_FC = 0.74), the core component of the ER translocon. Accessory translocation proteins were also upregulated to facilitate the efficient entry of nascent preproinsulin chains into the ER lumen, including *Ssr1* (log_2_FC = 0.51), *Srprb* (log_2_FC = 0.42), and *Srpr* (log_2_FC = 0.25).

This augmentation of secretory capacity building extended to the COPII vesicle components. The cargo selector *Sec24a* (log_2_FC = 0.91) was notably upregulated, alongside the structural outer coat units *Sec23a* (log_2_FC = 0.31) and *Sec13* (log_2_FC = 0.29). Simultaneously, increased expression of the proinsulin-processing enzyme *Pcsk1* (log_2_FC = 0.31) and remodeling of vesicle components ensured matching of trafficking flux. The core exocytosis factors for insulin granule docking and fusion were translationally elevated, including *Rab3a* (log_2_FC = 0.50), *Vamp2* (log_2_FC = 0.46), and *Snap29* (log_2_FC = 0.40). This transport was essentially supported by the upregulation of the cytoskeletal motor proteins such as *Kif5b* (log_2_FC = 0.65) and the dynein component *Dynll1* (log_2_FC = 0.58). Interestingly, the general endocytosis factors (*Rab2a*: log_2_FC = −0.61; *Rab14*: log_2_FC = −0.78; and *Rab5a*: log_2_FC = −0.77) were downregulated under acute high-glucose challenge. This bifurcation suggests that secretory cargo export takes precedence over membrane recycling to ensure efficient insulin secretion and resource allocation during GSIS (**Figure 4B**).

Regarding protein folding, while certain core chaperones were upregulated (*Hspa8*: log_2_FC = 1.16; *Dnajb1*: log_2_FC = 0.92; *Dnaja1*: log_2_FC = 0.72; *Erp44*: log_2_FC = 0.97; *Pdia3*: log_2_FC = 0.35), others remained unchanged or even suppressed (e.g., *Hspa5* and *Fkbp9*, with log_2_FC values of −0.22 and −0.67, respectively). This indicates a potential proteostatic imbalance, where the protein folding capacity fails to keep pace with the rapid expansion of the translation machinery, thereby increasing the risk of protein misfolding and ER stress (13–17) (**Figure 4C**).

### 3.5 High glucose reprograms signaling networks to drive an active secretory phenotype

The pivotal role of the mTOR pathway in coordinating nutrient sensing and metabolism is well established. Its dynamic post-translational phosphorylation is introduced in **Figure 2**. At the translational level, the expression of *Eif4b* increased approximately twofold (log_2_FC = 0.98), likely enhancing translation initiation by promoting ribosome loading onto mRNAs (18). Interestingly, the downregulation of *Eif4ebp1* (log_2_FC = −0.99) suggests a post-translational mechanism that relieves eIF4E from 4EBP1-mediated inhibition (19), thereby ensuring a rapid metabolic response and prioritized resource allocation. Concurrently, several scaffolding components were downregulated—including *Rragb* (log_2_FC = −0.79), which recruits mTORC1 to the lysosome for activation, and *Lamtor1*, *Lamtor3*, and *Lamtor4* (log_2_FC = −0.64, −0.60, and −0.69, respectively), which anchor the Rag GTPases to the lysosome, indicating a self-limiting negative feedback mechanism to prevent excessive mTOR activation (**Figure 4D**).

On the other hand, the cAMP signaling pathway was fine-tuned to sensitize beta cells to secretagogues. The transcription factor (TF) *Crem* (20), a key modulator of the cAMP response elements (CRE) crucial for insulin gene expression, increased over twofold (log_2_FC = 1.48). Two Gi-coupled receptors were downregulated under high-glucose conditions (*Npy2r*: log_2_FC = −1.39; *Oprm1*: log_2_FC = −1.00), relieving the inhibition of adenylate cyclase and boosting cAMP production. The Gs-coupled receptor *Taar1* was upregulated, further sensitizing the islets (log_2_FC = 0.97) (21). Intracellularly, the sharp downregulation of the cAMP-hydrolyzing enzyme *Pde1b* (log_2_FC = −2.03) likely sustains cAMP accumulation (**Figure 4D**).

### 3.6 Acute high-glucose treatment reprograms the translational landscape of metabolic genes in pancreatic islets

#### 3.6.1 Mitochondria

**Figure 5:**
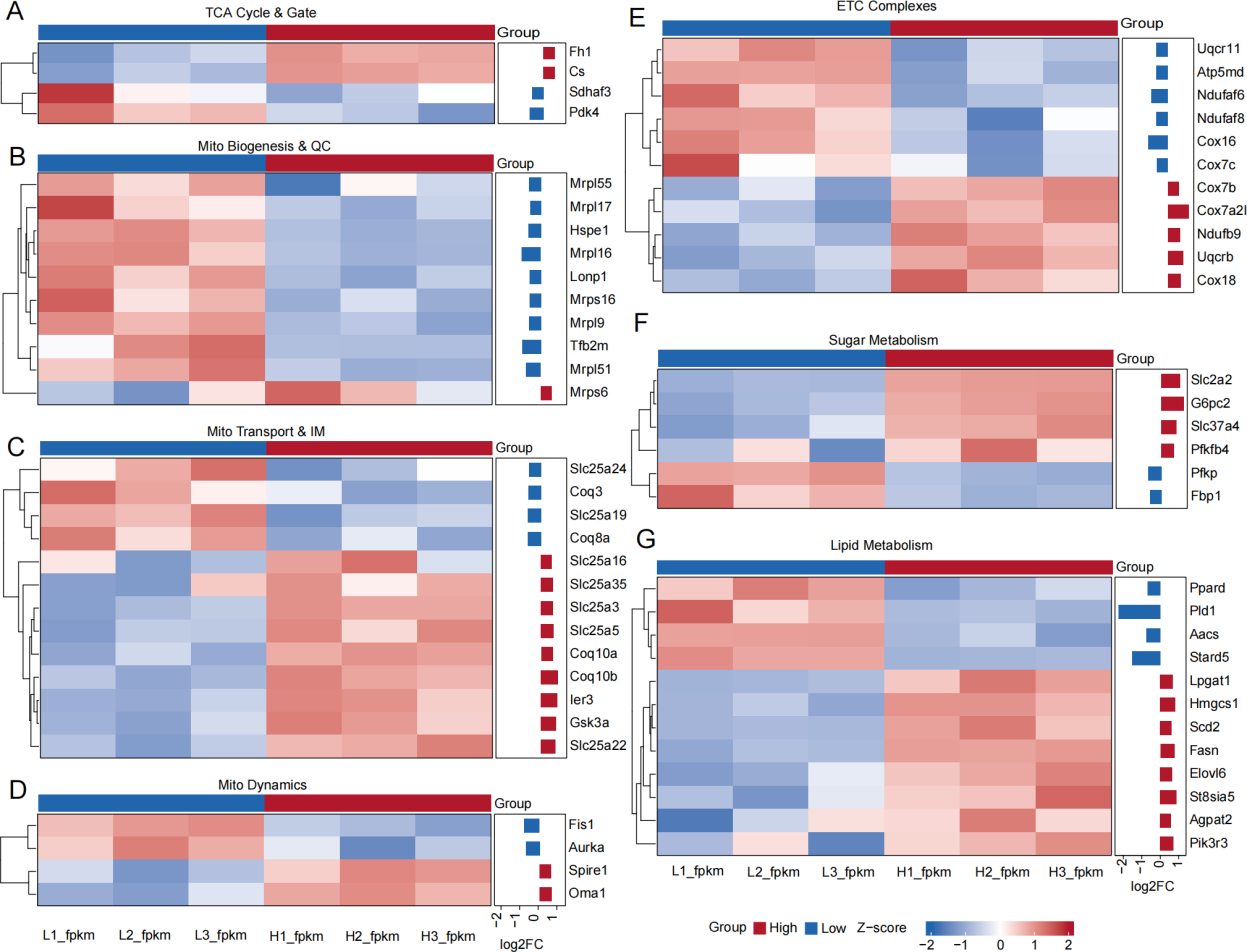
Translational regulation of metabolism. Heatmap of genes in pathways associated with the tricarboxylic acid (TCA) cycle and gate **(A)**, mitochondrial biogenesis and QC (quality control) **(B)**, mitochondrial transport and inner membrane (IM) **(C)**, mitochondrial dynamics **(D)**, electron transport chain (ETC) complexes **(E)**, sugar metabolism **(F)**, and lipid metabolism pathway **(G)**. The color gradient of each gene corresponds to its scaled *Z* score. Bar length reflects the log_2_FC.

Within the mitochondrial matrix, we observed a robust translational induction of the core tricarboxylic acid (TCA) cycle enzymes (*Cs* and *Fh1*: log_2_FC = 0.61 and 0.60, respectively). On the other hand, the translation of *Pdk4* (log_2_FC = −0.74), a primary inhibitor of the pyruvate dehydrogenase complex (PDC), was suppressed, favoring the molecular opening of the mitochondrial gate to facilitate the efficient conversion of pyruvate to Acetyl-CoA (**Figure 5A**). Paradoxically, mitochondrial biogenesis appears to be inhibited at the translational level. In contrast to cytosolic ribosomes, many genes encoding mitochondrial ribosomal proteins were downregulated (*Mrpl16*: log_2_FC = −1.03; *Mrpl51*: log_2_FC = −0.79; *Mrpl55*: log_2_FC = −0.64; *Mrpl9*: log_2_FC = −0.63; *Mrpl16*: log_2_FC = −1.03; *Mrpl17*: log_2_FC = −0.59), alongside the reduction of *Mtg2* (log_2_FC = −0.84), a key factor in mitochondrial translation and ribosome assembly (**Figure 5B**).

Our data revealed a divergence in translation between the genes encoding the inner (IM) and outer membranes (OM) under high glucose. Two genes encoding the key enzymes for ubiquinone biosynthesis in the IM (*Coq8a* and *Coq3*: log_2_FC = −0.72 and −0.70, respectively) were downregulated, while OM-associated metabolite transporters were upregulated (*Slc25a5/AntT2*: log_2_FC = 0.69; *Gsk3a*: log_2_FC = 0.82; *Ier3*: log_2_FC = 0.87) (**Figure 5C**).

Acute high glucose also affected the mitochondrial dynamics-related genes. We observed a significant induction of *Oma1* (log_2_FC = 0.64) and *Spire1* (log_2_FC = 0.63), which function in fragmentation and crista remodeling. In contrast, the translation of *Fis1* (log_2_FC = −0.84) and *Aurka* (log_2_FC = −0.75) was downregulated, likely serving as a negative feedback in the case of excessive organelle degradation. This coordinated shift in mitochondrial gene translation might optimize the mitochondrial surface area for ATP exchange during the transition to a highly secretory state (**Figure 5D**).

Overall, the mitochondria prioritize leveraging existing components over expanding the organelle volume to maximize the ATP output. This strategy allows efficient resource allocation and helps prevent excessive oxidative stress.

#### 3.6.2 Glucose

*Slc2a2*, which encodes the primary glucose transporter GLUT2, was strongly upregulated (log_2_FC = 1.00), reinforcing the molecular basis for rapid glucose sensing and uptake under high-glucose conditions. Concurrently, acute high-glucose treatment targets the G6P cycle to modulate the glycolytic flux at the translational level, which prioritizes glycogen storage. This was evidenced by the upregulation of *G6pc2* (log_2_FC = 1.22), a rate-limiting enzyme in gluconeogenesis and glycogenolysis (22), and *Slc37a4* (log_2_FC = 0.81), a G6P transporter (23), alongside the downregulation of *Fbp1* (log_2_FC = −0.62), a rate-limiting gluconeogenesis enzyme (24). The downregulation of *Pfkp* (log_2_FC = −0.71), a rate-limiting glycolysis enzyme (25), and the upregulation of its allosteric activator *Pfkfb4* (log_2_FC = 0.69) (26) suggest an adaptation to prevent excessive glycolytic overflow and potentially shunt the G6P towards storage and the pentose phosphate pathway (PPP) to generate NADPH for downstream lipogenesis (**Figure 5F**).

#### 3.6.3 Lipid

Acute high glucose shifts the lipid from catabolism towards anabolism, as evidenced by the significant upregulation of *Fasn* (log_2_FC = 0.77), a rate-limiting enzyme for long-chain fatty acid synthesis, and *Hmgcs1* (log_2_FC = 0.81), a rate-limiting enzyme in cholesterol synthesis, and a reciprocal repression of *Aacs* (log_2_FC = −0.74), which is involved in ketone body utilization, and *Ppard* (log_2_FC = −0.68), a key transcriptional regulator of fatty acid oxidation. However, despite increased cholesterol biosynthesis, its intracellular distribution might be compromised by the sharp downregulation of *Stard5* (log_2_FC = −1.52) (27). Furthermore, the phospholipid landscape was remodeled (*Agpat2*: log_2_FC = 0.59; *Lpgat1*: log_2_FC = 0.67; *St8sia5*: log_2_FC = 0.89; *Pld1*: log_2_FC = −2.27) (28) to provide the essential membranous framework for vesicle trafficking and intracellular signaling (*Pik3r3*: log_2_FC = 0.73; *Pik3r2*: log_2_FC = −0.80) (29, 30) (**Figure 5G**).

### 3.7 High glucose reprograms the translational landscape of transcription factors and epigenetic regulators

**Figure 6:**
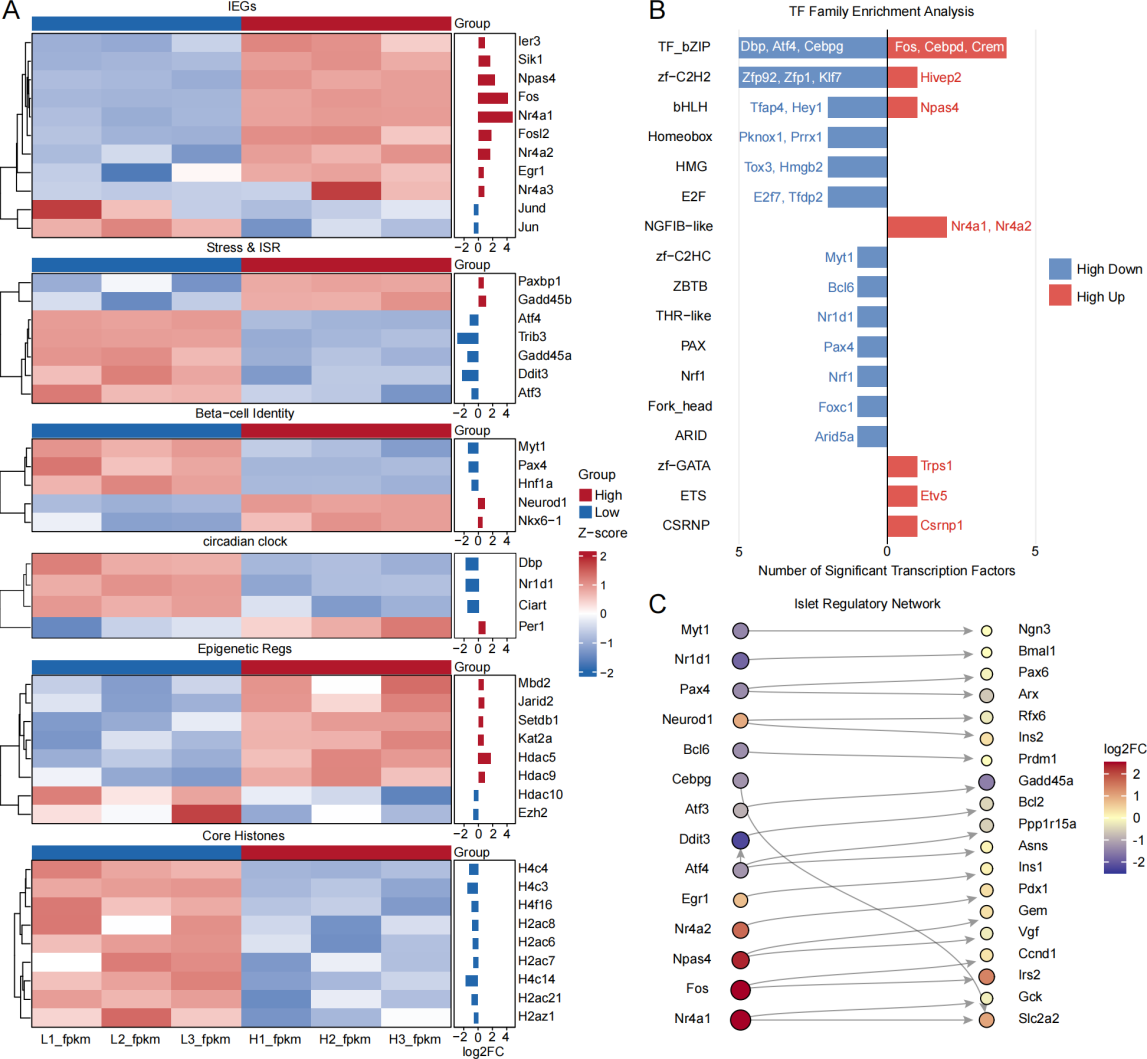
Translational regulation of transcription factors (TFs) and epigenetic regulatory factors. **(A)** Heatmap of the genes in pathways associated with immediate early genes (IEGs) (Imm *Early*), stress and integrated stress response (ISR), beta-cell identity, circadian clock, epigenetic regulation, and core histones. Red indicates upregulation, while blue indicates downregulation. The color gradient of each gene corresponds to its scaled *Z*-score. Bar length reflects the log_2_FC. **(B)** Bidirectional bar chart quantifying the number of upregulated (red) and downregulated (blue) genes across the top 10 glucose-responsive TF families. **(C)** Islet regulatory network (no-overlap version) of TFs and their key downstream targets. The color of each bubble represents the log_2_FC.

Our Ribo-seq data identified 135 TFs with significant altered translation by acute high glucose, comprising 49 upregulated and 86 downregulated genes (**Figure 6A**). The 10 most affected TF families were *zf-C2H2*, *TF-bZIP*, *Homeobox*, *bHLH*, *ZBTB*, *HMG*, *ETS*, *THR-like*, *MBD*, and *NGFIB-like* (**Figure 6B**), forming a regulatory network that orchestrates the key downstream targets involved in insulin secretion, glucose sensing, and cellular stress responses (**Figure 6C**; **Supplementary Table 3**).

Beyond the IEGs and stress-related factors, the transcriptional network governing β-cell identity underwent significant remodeling. Specifically, *Neurod1* (log_2_FC = 0.94) and *Nkx6-1* (log_2_FC = 0.61) were upregulated, whereas *Myt1* (log_2_FC = −1.43), *Hnf1a* (log_2_FC = −0.93), *Tox3* (log_2_FC = −1.26), and *Pax4* (log_2_FC = −1.36) were downregulated, reflecting a mature β-cell program under high glucose. However, acute high-glucose exposure disturbed the expression profile of the circadian clock genes, indicated by the sharp suppression of *Nr1d1* (log_2_FC = −1.84), *Dbp* (log_2_FC = −1.77), and *Ciart* (log_2_FC = −1.56), alongside the notable induction of *Per1* (log_2_FC = 0.91).

Furthermore, our data revealed a condition of comprehensive epigenetic remodeling and potential chromatin instability. The broad downregulation of multiple core H4/H2A histones (e.g., *H4c3*: log_2_FC = −1.46; *H4c14*: log_2_FC = −1.70; and *H2ac8*: log_2_FC = −0.83) implies a reduced demand for *de novo* nucleosome assembly, resulting in a more relaxed and accessible chromatin structure for rapid transcriptional reprogramming (31). This is further illustrated by the translational profiles of epigenetic regulators, including the methyl-CpG reader *Mbd2* (log_2_FC = 0.74), the methyltransferase *Setdb1* (log_2_FC = 0.65), the acetyltransferase *Kat2a* (log_2_FC = 0.75), and the histone deacetylases (e.g., *Hdac5*: log_2_FC = 1.74; *Hdac9*: log_2_FC = 0.90; and *Hdac10*: log_2_FC = −0.60). The polycomb repressive complex 2 (PRC2) showed reciprocal changes in its catalytic subunit *Ezh2* (log_2_FC = −0.72) and the regulatory subunit *Jarid2* (log_2_FC = 0.78) (32).

Finally, the upregulation of *Polr1e* (a subunit of RNA polymerase I that is exclusively responsible for rRNA transcription: log_2_FC = 1.15) consistently aligned with the global surge in ribosome biogenesis in high glucose (33).

### 3.8 Translation efficiency analysis revealed a multilayered regulation mechanism under high glucose

**Figure 7:**
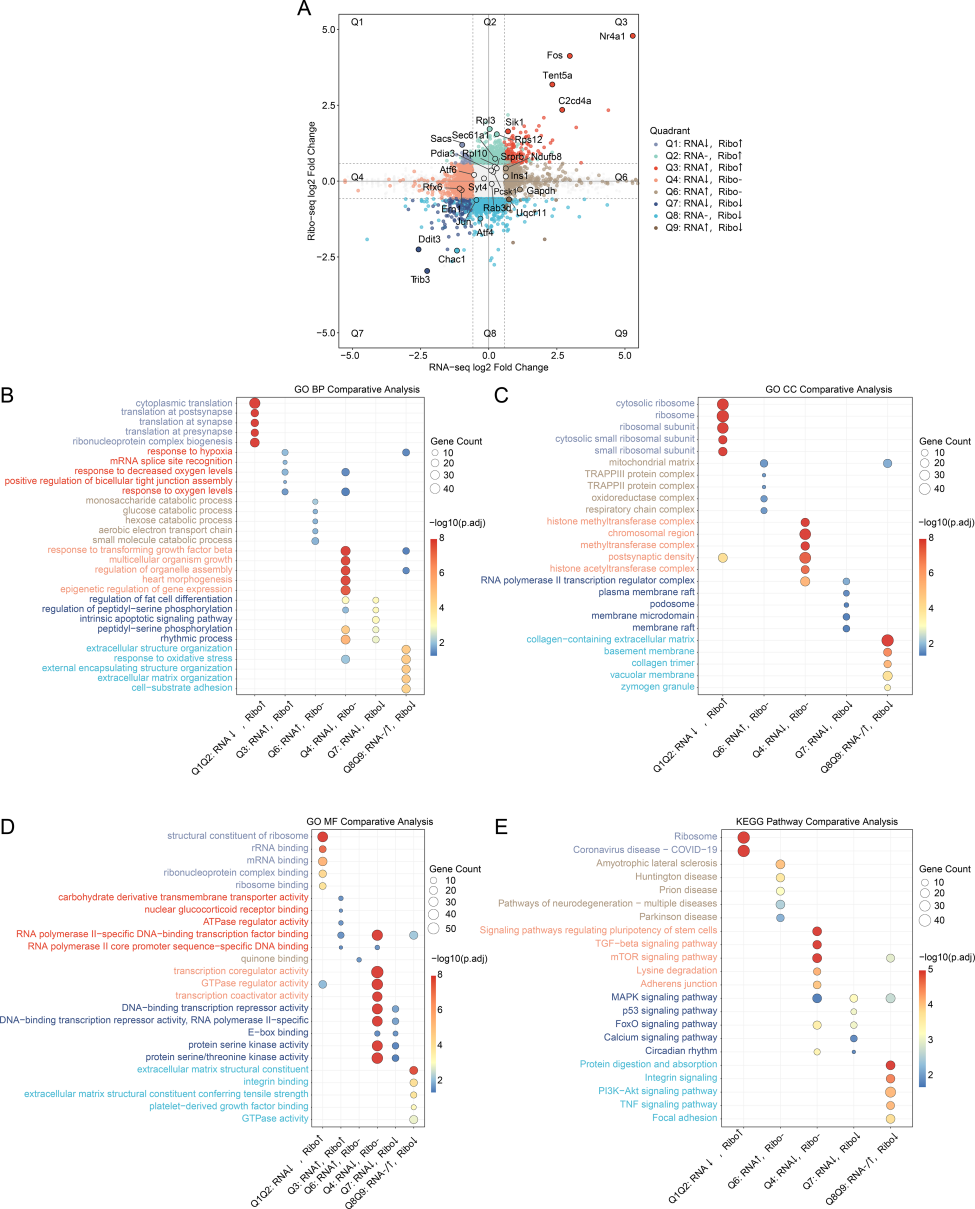
Gene Ontology (GO) and Kyoto Encyclopedia of Genes and Genomes (KEGG) enrichment analysis of the genes in each quadrant representing translation efficiency. **(A)** Nine-quadrant plot illustrating the classification of the differentially expressed genes according to the log2FC values derived from RNA-seq and Ribo-seq. **(B–D)** Bubble plots showing significantly enriched GO categories (biological process, cellular component, and molecular function) among quadrant-defined gene subsets. **(E)** Bubble plots showing significantly enriched KEGG pathways among quadrant-defined gene subsets. The color of each bubble represents the adjusted −log10 (padj.), and the size of the bubble corresponds to the gene count.

To investigate the translational control of the core DEGs, we integrated Ribo-seq and bulk RNA-seq data to calculate the TE and visualized the regulatory landscape in a nine-quadrant plot (**Figure 7A**). Genes showing a significant change (|log_2_FC| > 0.58, *p* < 0.05) in either the Ribo-seq or the RNA-seq dataset were subjected to functional enrichment analysis (**Figures 7B–E**; **Supplementary Table 4**).

Many classical IEGs and metabolic hubs were localized in Q3 (upper right), including *Nr4a1*, *Fos*, and the T2DM-susceptibility gene *C2cd4a* (34). The coordinated increase in both transcript abundance and ribosome occupancy suggests a synergistic and multilayered mobilization to prioritize high-flux protein production. Conversely, a number of stress-related genes including *Trib3* clustered in Q7, indicating a doubly safeguarded repression. The integrated stress response (ISR) master regulator *Atf4*, along with *Jun* and *Mapk14*, was located in Q8 and was directly silenced at the translational level.

Genes related to cytosolic translation were found in Q2, including several ribosomal subunits, elongation factors, and translational regulators (e.g., *Pabpc4*, *Pabpc1*, *Ago2*, and *Zc3h12c*). The self-expansion of the translational machinery is driven primarily by translational enhancements, which enables a rapid response to acute high glucose. Furthermore, we identified several survival-critical genes with a robust increased TE despite stable or declining mRNA levels in Q1/Q2/Q4, such as the beta-cell identity factor *Rfx6*, the survival kinase *Hipk2*, the insulin signaling component *Irs2*, and the chaperone *Sacs*. This prioritization potentially serves as a cytoprotection to bolster metabolic stress resilience while preserving cellular identity and function.

Interestingly, the diabetic marker *Tnxip*, a negative regulator of glucose uptake and a promoter of oxidative stress, displayed a marked decrease in TE. Although its transcription was highly induced, its ribosome occupancy increased disproportionately less. This translational buffering acts as a post-transcriptional checkpoint, likely counteracting overwhelming oxidative stress. A similar mechanism may govern the genes in Q6/Q8/Q9, such as the mitochondrial ribosomal proteins, the respiratory chain component (*Uqcr11*), the core metabolic enzymes (*Gapdh* and *Atp5d*), and the redox-sensitive regulator *Txn1*. Cells maintained or increased the mRNA standby inventory while suspending their translation. It serves as a metabolic bottleneck to limit the energetic burden of organelle biogenesis and actively prevent mitochondrial overactivation or reactive oxygen species (ROS) bursts during metabolic surges.

## 4 Discussion

The core of pancreatic β-cell function lies in its ability to mount a rapid and precise response to glucose fluctuations. Although the transcriptional impact of glucose on islets has been extensively studied, our Ribo-seq data provide the precise TE at a genome-wide scale and uncovered the translation kinetic signatures. It reveals that acute high glucose triggers an immediate, profound, and coordinated reprogramming of the translational landscape.

Mechanistically, the increased ATP/ADP ratio in high glucose suppresses AMPK activity, thus allowing for mTORC1 activation and licensing translation (10). Ribosomal proteins and elongation factors are encoded by the canonical 5′-terminal oligopyrimidine (5′TOP) mRNAs, defined by a specific motif at their 5′-end: a cytidine (C) immediately after the cap, followed by a stretch of 4–15 pyrimidines (C or T). Recent studies have shown that LARP1 (La-related protein 1) binds TOP mRNAs to repress their translation when mTORC1 is inactive (35). Upon glucose stimulation, mTORC1 phosphorylates LARP1, relieving this repression and permitting the translational surge of TOP mRNAs. This represents a feed-forward mechanism to sustain translational capacity. In contrast, the majority of initiation factors lack this motif, thus exhibiting a divergent regulation. Beyond accelerating the elongation speed, high glucose coordinately upregulates the ribosome-associated quality control to ensure translational fidelity. Concurrently, our Ribo-seq data highlight a potential novel regulatory axis in glucose homeostasis through the modulation of mRNA stability. This is evidenced by the synchronized induction of *Tenta5* (also known as *Fam46a*), *Pabpc1*, and *Pabpc4*, which might enhance TE by extending the poly(A) tails of specific secretory mRNAs.

Acute high glucose does not only modulate protein synthesis rates, but it also triggers a qualitative shift in the translational panel. The most striking phenotype is the robust induction of cytoprotective IEGs. For example, the orphan nuclear receptors *Nr4a1* and *Nr4a2* drive the transcription of the genes critical for metabolic plasticity, allowing beta cells to sustain high protein synthesis loads without undergoing apoptosis. Similarly, the calcium-dependent induction of *Npas4* integrates the insulin secretion with survival-promoting gene expression (36), while the AP-1 complex components (*Fos* and *Fosl2*) support β-cell proliferation and long-term adaptation (37). Conversely, the translation of the stress-related genes (e.g., *Ddit3*, *Trib3*, *Atf4*, and *Atf3*) is nearly eliminated within 2 h of high-glucose treatment. Together with eIF2α dephosphorylation, this suggests that the protein synthesis arrest is alleviated.

Our Western blotting results confirmed a dramatic increase in the preproinsulin and proinsulin protein levels after 2 h in high glucose, while the Ribo-seq data showed only a modest increase in *Ins1* (11%) and *Ins2* (27%) translation. One simple explanation is the high abundance of insulin mRNA; thus, even a moderate increase in the ribosome flux could yield a substantial protein production. Moreover, this discrepancy may be explained by a kinetic acceleration model. Since Ribo-seq captures a static snapshot of the ribosome positions, the extent of the translation regulation might be underestimated when the elongation rate is modulated. Our data, including the rapid dephosphorylation of eEF2 and the dose-dependent scaling of proinsulin, suggest a favorable environment for translation elongation under acute glucose stimulation. A more in-depth study of the translation dynamics is warranted.

In addition, we observed a prominent accumulation of ribosome footprints at the coding sequence corresponding to AA 56–72 of preproinsulin-1. Because the preproinsulin signal peptide is 24 residues in length and the ribosomal exit tunnel accommodates approximately 30–40 AA, this peak is positionally consistent with the site of signal recognition particle (SRP)-mediated elongation arrest upon emergence of the signal peptide (38). Under high-glucose stimulation, this ribosome occupancy peak is significantly diminished compared with the downstream coding region, suggesting a relief of translational pausing. Our Ribo-seq analysis also uncovered a synchronized translational upregulation of the translocon components (e.g., *Sec61a1* and *Srprb*) (39–41) and the downstream secretory machinery. The cargo selector *Sec24a* was specifically upregulated, suggesting a specialized function in the selective transport of proinsulin. Concurrent upregulation of the outer coat components (*Sec31a* and *Sec13*) and the processing enzyme *Pcsk1*, although to a lesser extent, ensures a concerted expansion of the secretory pathway to sustain insulin production.

During the insulin secretion surge in the acute phase, the mitochondria appear to prioritize a cost-effective “efficiency-over-expansion” strategy that favors increased ATP production capacity from existing machinery. High glucose induces a coordinated translational reprogramming of metabolic genes, characterized by the induction of the TCA cycle enzymes (e.g., *Cs* and *Fh1*) and the metabolite transporters (e.g., *Slc25a5* and *Gsk3a*) alongside the reciprocal suppression of the mitochondrial ribosomal proteins. Such a strategy is consistent with an optimization to meet the metabolic demand while limiting the proteostatic burden of energy-intensive biogenesis. Another example of the adaptive logic is the reduced translation of the key mTORC1 scaffolding components, such as *Rragb* and *Lamtor1/3/4*, which might serve as an adaptive dampening mechanism to prevent the overactivation of the mTOR pathway. Concurrently, the translational downregulation of *Eif4ebp1* (encoding the eIF4E inhibitor 4E-BP1) suggests a dynamic and multilayered regulatory mechanism.

From the perspective of protein metabolism, despite the robust upregulation of the translation machinery and secretory cargoes, ER-resident chaperones (e.g., *Hspa5*, *Calr*, and *Pdia*) did not keep pace accordingly. In contrast, the upregulation of *Hspa8*, *Dnaja1*, and *Dnajb1* suggests that the cytosolic folding system acts as a primary buffer to prevent the aggregation of newly synthesized chains before ER translocation (13, 16). Furthermore, high glucose appears to rewire the degradation system. *Bag2* and *Bag3* were significantly upregulated, potentially stabilizing nascent proteins by inhibiting CHIP-mediated ubiquitination (42).

Our TE analysis identified a group of genes that undergo robustly upregulated translation despite stable or declining mRNA levels. This translational prioritization is thought to exert a cytoprotective effect, potentially enhancing metabolic stress resilience to preserve β-cell identity and function. In contrast, several genes exhibited a significant decrease in TE even when their mRNA pool remained stable or increased, with notable examples including *Txnip* and various mitochondrial genes. This strategy likely functions as a translational buffer to mitigate oxidative stress during the metabolic surge.

There are still limitations to the current research that warrant discussion. Firstly, as insulin biosynthesis is a highly dynamic process, our Ribo-seq snapshot at a single time point captures a specific window of the acute response. Future Ribo-seq studies involving more time points and varying glucose concentrations would provide higher temporal resolution and broader physiological relevance. Secondly, the discrepancies between translation and protein abundance highlight the need to integrate translatomics with proteomics, and the role of protein stability remains to be addressed. Further functional studies are also needed to delve deeper into the critical mechanisms, such as mitochondrial remodeling and SRP-mediated arrest and post-translational regulation. Furthermore, exploring the translational atlas in diabetic mouse models under chronic hyperglycemia is warranted to understand how these adaptive mechanisms fail over time. Finally, future work will also focus on correlating specific regulatory sequence features with the translational behaviors observed in this study.

In summary, acute high glucose reprograms the translational landscape of islets, shifting the cell from a stress/repressing state to an adaptive/secretory phenotype. This study identifies a repertoire of glucose-responsive targets. Ultimately, it lays the groundwork for the development of therapeutic strategies to combat beta-cell failure in T2DM.

## Data Availability

The data presented in the study are deposited in the GEO repository, accession numbers GSE334681 and GSE334569.
